# Supplementary material for: All-optical complex field imaging using diffractive processors
Source: Light Sci Appl. 2024 May 28;13:120. doi: 10.1038/s41377-024-01482-6 (PMC11130282; doi:10.1038/s41377-024-01482-6)
Supplement: Supplementary file 1 — Supplementary Material [file 41377_2024_1482_MOESM1_ESM.pdf]

Supplementary Materials for

All-optical complex field imaging using diffractive processors

Jingxi Li[1,2,3], Yuhang Li[1,2,3], Tianyi Gan[1,3], Mona Jarrahi[1,3], and Aydogan Ozcan[1,2,3*]

[1]Electrical and Computer Engineering Department, University of California, Los Angeles, CA, 90095, USA

[2]Bioengineering Department, University of California, Los Angeles, CA, 90095, USA

[3]California NanoSystems Institute (CNSI), University of California, Los Angeles, CA, 90095, USA

[*]Correspondence to: ozcan@ucla.edu

Supplementary Figures

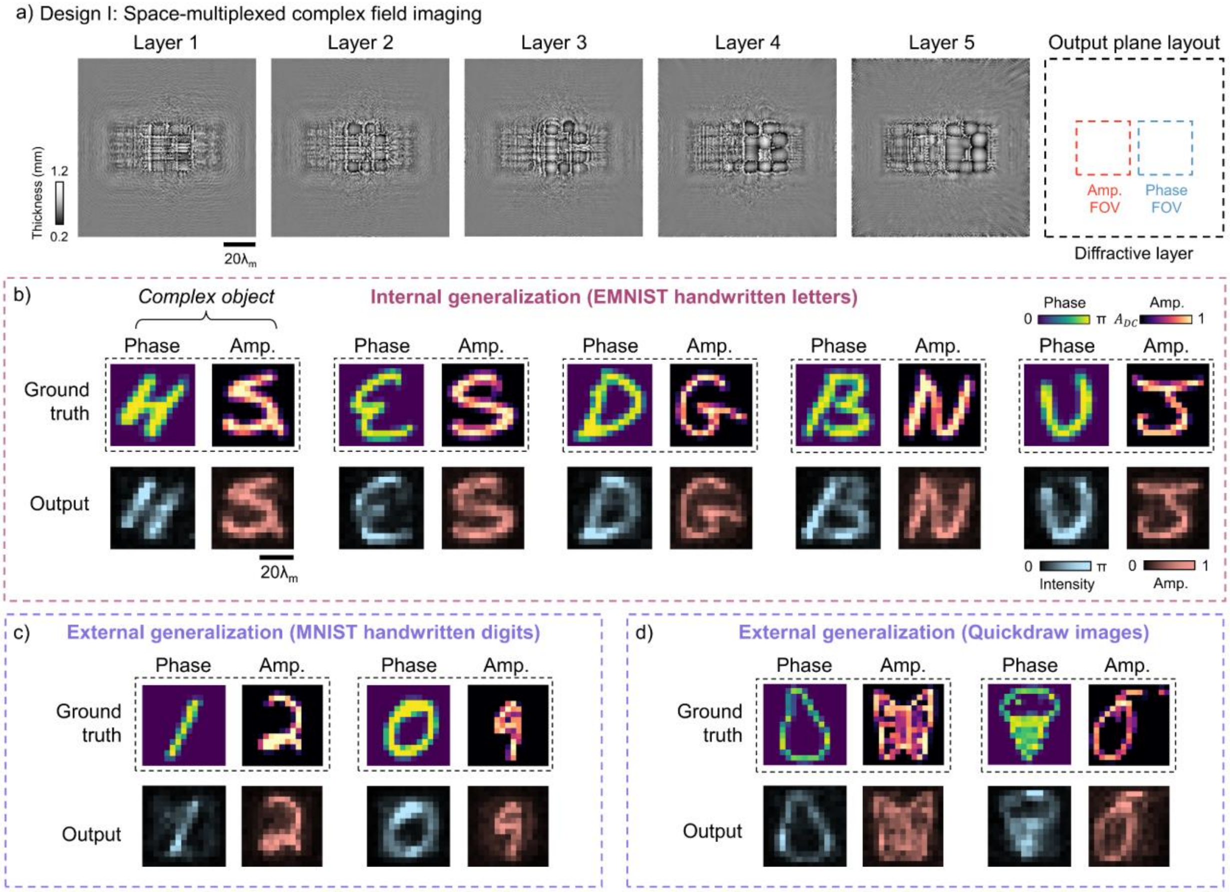

Figure S1. Blind testing results of the diffractive complex field imager using design I. Same as Fig. 2 of the main text, except that the output amplitude and phase images from this diffractive imager model are measured using the same wavelength.

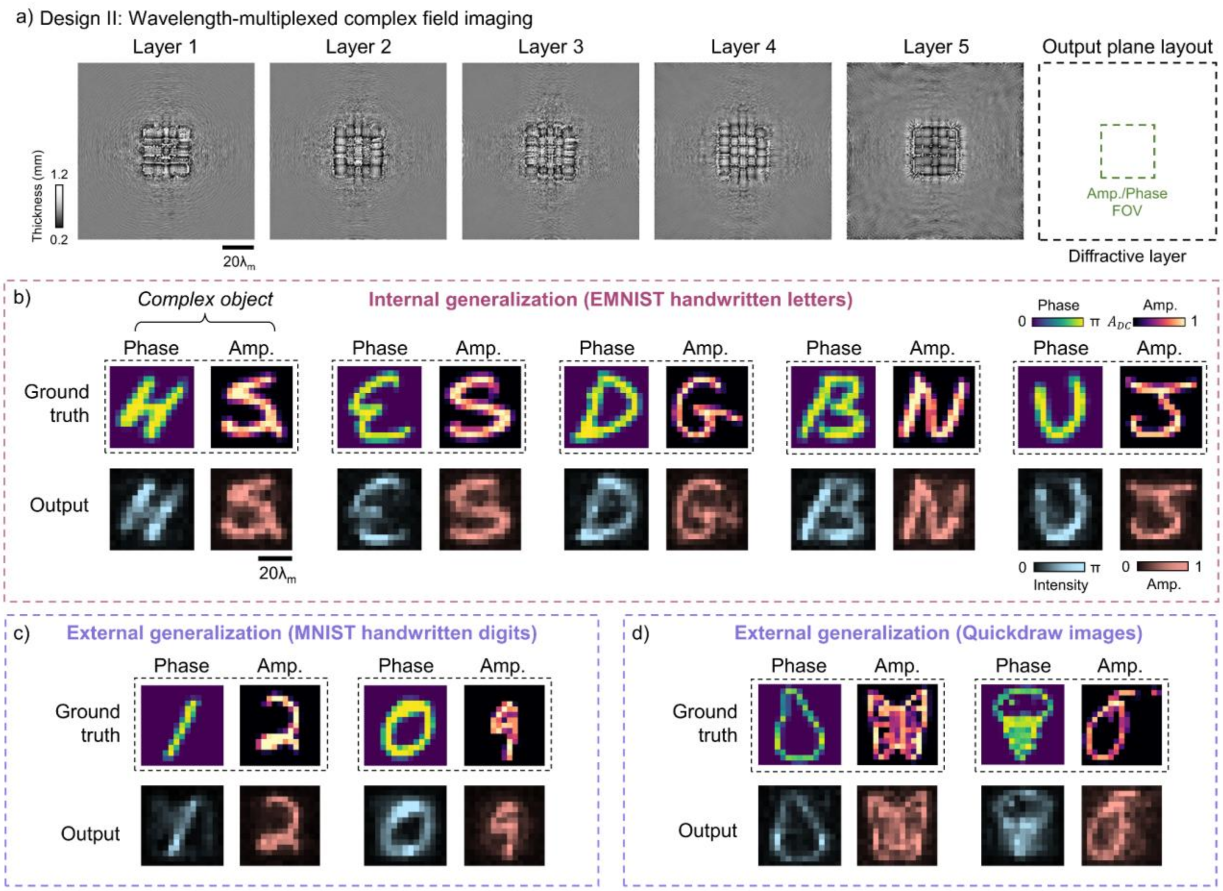

Figure S2. Blind testing results of the diffractive complex field imager using design II. Same as Fig. 2 of the main text, except that the output amplitude and phase images from this diffractive imager model are measured using a single common output FOV.

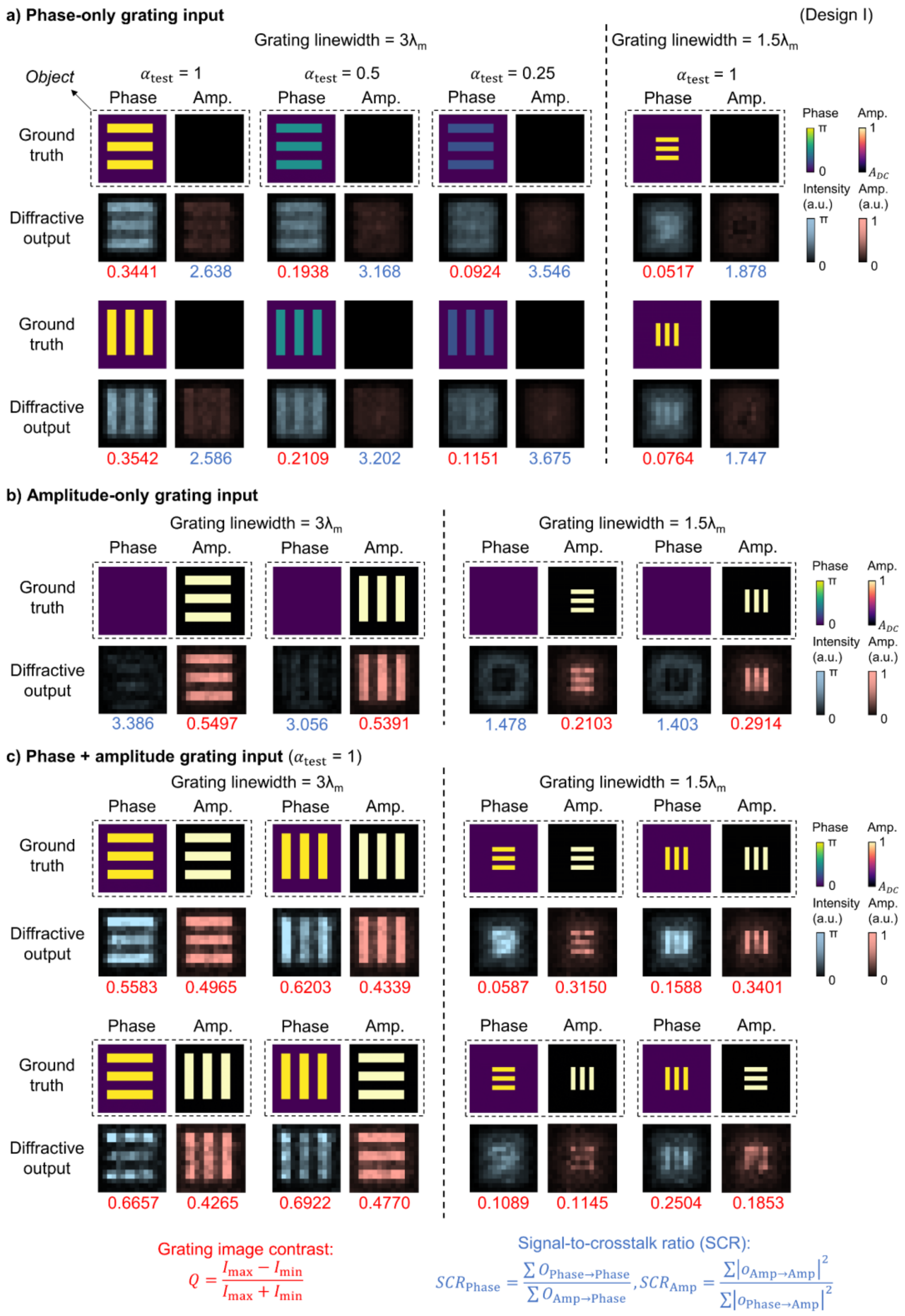

Figure S3. Performance analysis of the diffractive complex field imager model shown in Supplementary Fig. S1. Same as Fig. 3 of the main text, except that the output amplitude and phase images from this diffractive imager model are measured using the same wavelength.

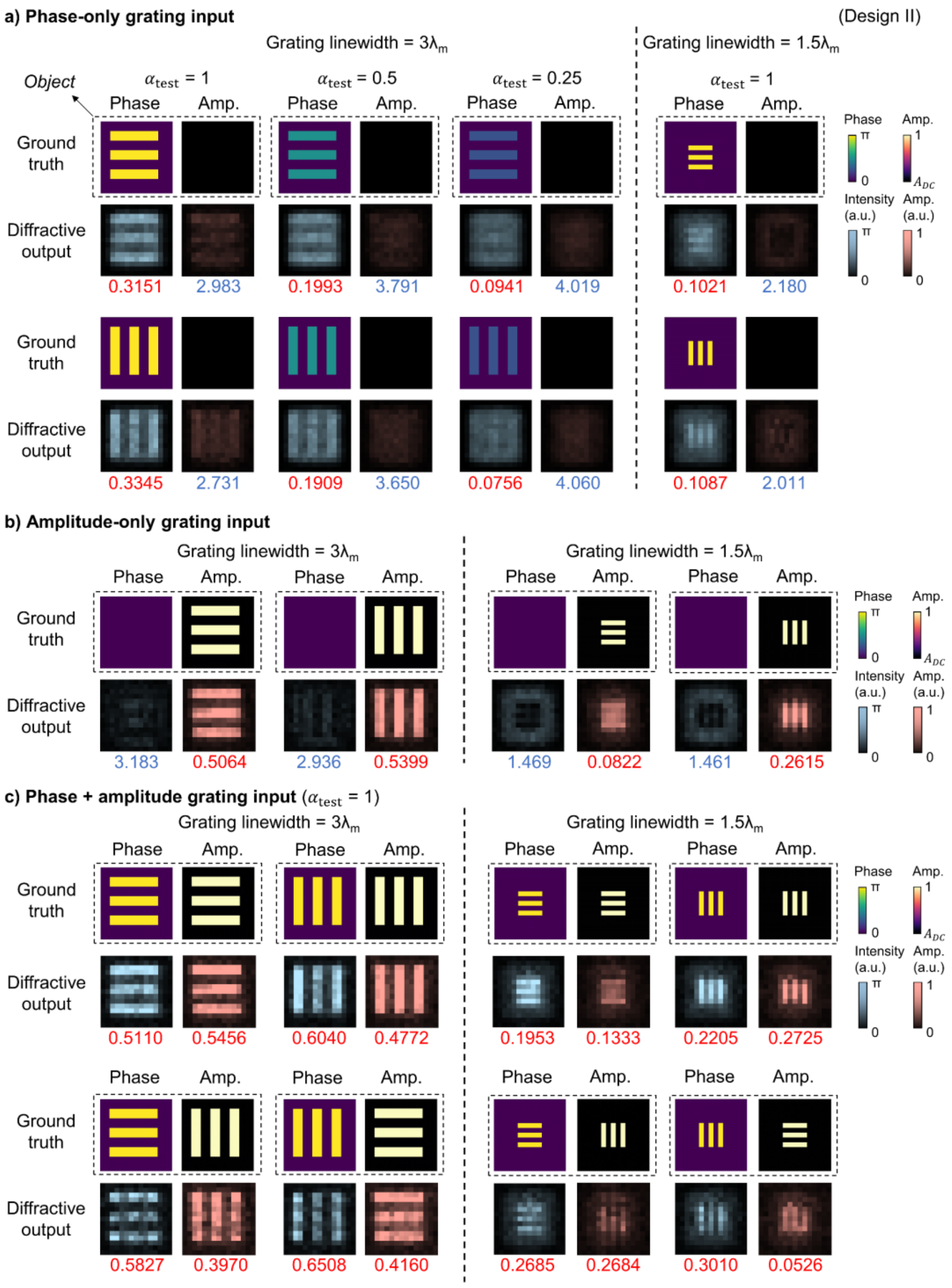

Grating image contrast:

$$Q = \frac{I_{\max} - I_{\min}}{I_{\max} + I_{\min}}$$

Signal-to-crosstalk ratio (SCR):

$$SCR_{\text{Phase}} = \frac{\sum o_{\text{Phase}\to\text{Phase}}}{\sum o_{\text{Amp}\to\text{Phase}}}, SCR_{\text{Amp}} = \frac{\sum |o_{\text{Amp}\to\text{Amp}}|^2}{\sum |o_{\text{Phase}\to\text{Amp}}|^2}$$

Figure S4. Performance analysis of the diffractive complex field imager model shown in Supplementary Fig. S2. Same as Fig. 3 of the main text, except that the output amplitude and phase images from this diffractive imager model are measured using a single common output FOV.

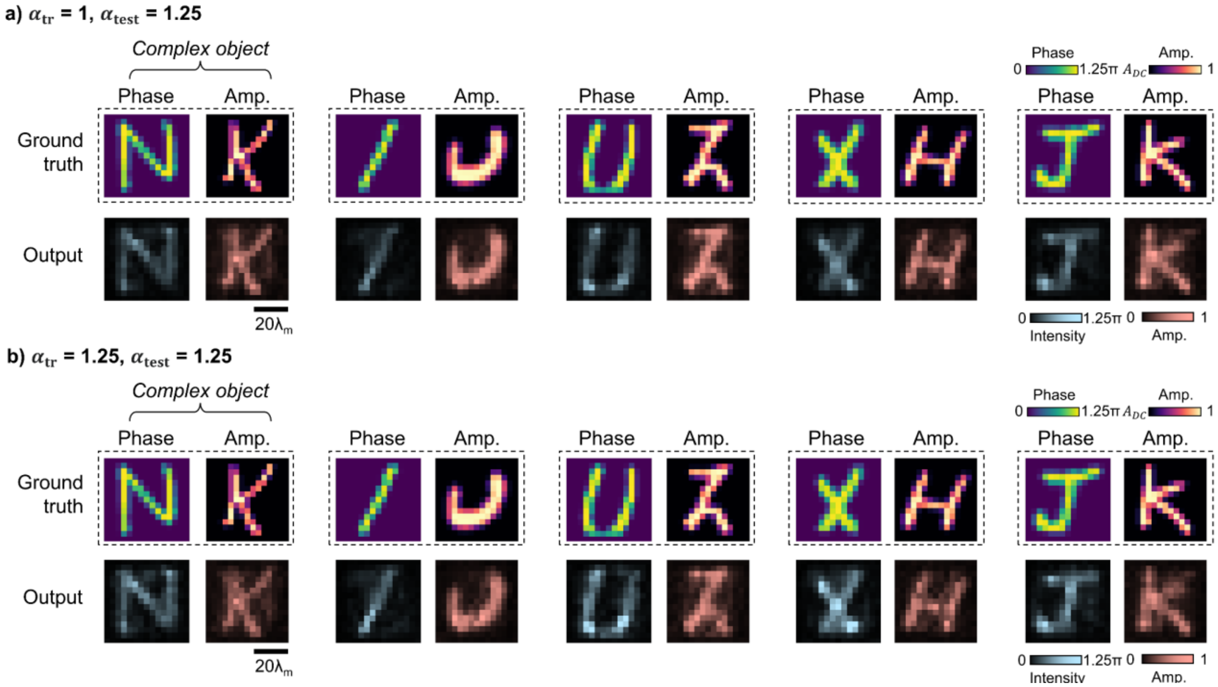

Figure S5. Analysis of the impact of the input phase contrast on the imaging performance of diffractive complex field imagers. Same as Fig. 4 of the main text, except that the phase contrast parameter used in the testing phase (α_{test}) is set as 1.25, and the diffractive model in (b) was trained using α_{tr} = 1.25.

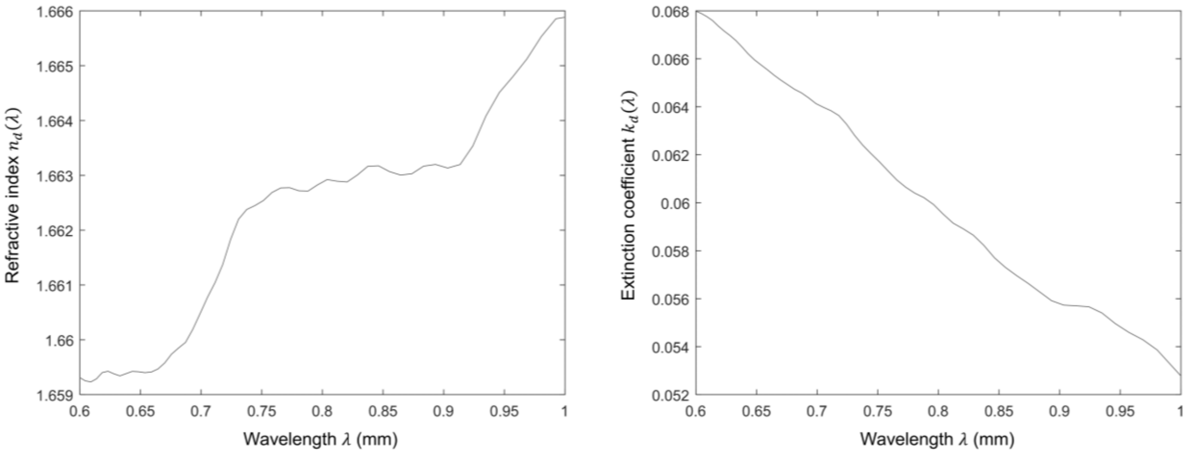

Figure S6. Dispersion curves of the material used for the 3D-fabricated diffractive layers, including the refractive index $n_d(\lambda)$ (left) and the extinction coefficient $k_d(\lambda)$ (right).
